# Construction of prognostic signature of patients with oral squamous cell carcinoma based on pyroptosis-related long non-coding RNAs

**DOI:** 10.3389/fsurg.2022.935765

**Published:** 2022-08-23

**Authors:** Yuqi Xin, Jieyuan Zhang, Qingkun Jiang, Jiaxuan Qiu

**Affiliations:** ^1^ Department of Stomatology, The First Affiliated Hospital of Nanchang University, Nanchang, China; ^2^ Medical College, Nanchang University, Nanchang, China

**Keywords:** oral squamous cell carcinoma, pyroptosis, long non-coding RNAs, signature, survival, prognosis

## Abstract

**Background and objective:**

Oral squamous cell carcinoma (OSCC) is the most common malignant tumor in the head and neck, and its morbidity and mortality are increasing year by year. Changes in key genes are thought to be closely related to the occurrence and development of OSCC. Pyroptosis is an inflammatory form of programmed cell death that has been implicated in malignancies and inflammatory diseases. Changes in the expression of long noncoding RNAs may also affect tumorigenesis and progression. In this study, our main objective was to evaluate the association between pyroptosis-related lncRNAs and prognosis in patients with OSCC.

**Methods:**

The RNA-seq data and clinicopathological data of OSCC patients are from The Cancer Genome Atlas database. The pyroptosis gene set is obtained from Gene Set Enrichment Analysis database. Univariate COX, Lasso and multivariate COX regression analyses were used for the construction of risk prognostic models of OSCC, eight lncRNAs were incorporated into prognostic models. The Kaplan-Meier method and log-rank test were used to evaluate the differences of overall survival between patients in high-risk and low-risk groups. The reliability of predictions across the dataset was analyzed by receiver operating characteristic (ROC) curves. The immune signature score was calculated using the single-sample gene set enrichment analysis.

**Results:**

Eight pyroptosis-related lncRNAs were used to construct prognostic signature of OSCC, including AC136475.2, AC024075.2, JPX, ZFAS1, TNFRSF10A-AS1, LINC00847, AC099850.3 and IER3-AS1. According to this prognostic signature, patients with OSCC were divided into high-risk and low-risk groups. Kaplan-Meier survival analysis showed that the survival rate of the high-risk group was significantly lower than the low-risk group. ROC area for risk score was 0.716, and ROC area of the 8 lncRNAs are all between 0.5 and 1, implied that these lncRNAs had high accuracy in predicting the prognosis of OSCC patients. Immune Infiltration findings suggested that these lncRNAs affected immune responses in the microenvironment of OSCC.

**Conclusion:**

The prognostic signature based on pyroptosis-related lncRNAs potentially serves as an independent prognostic indicator for OSCC patients. And this signature facilitates research on targeted diagnosis and treatment of patients diagnosed with OSCC.

## Introduction

Head and neck squamous cell carcinoma (HNSCC) is the sixth most common malignant tumor globally (1). Oral squamous cell carcinoma (OSCC) is a group of heterogeneous cancers that originate in the oral cavity, and is the main type of HNSCC (2). Over the past few years, the morbidity and mortality of OSCC have increased significantly, and the 5-year survival rate of patients is poor, less than 60% (3–5).Drinking and smoking are the most recognized factors for OSCC (6). In addition, viruses and microorganisms are closely related to the development of OSCC, such as Candida albicans, human papilloma virus (HPV) and Epstein-Barr virus (EBV) (7–9). Recent studies have shown that in addition to these recognized risk factors, the development of OSCC is closely related to changes in key genes (10). Currently, there are no effective biomarkers for the early diagnosis and prognosis prediction of oral cancer. Therefore, it is necessary to identify new biomarkers for the early diagnosis of OSCC and predict the prognosis of OSCC.

wCaspase 1-dependent programmed cell death (also known as pyroptosis) is a form of inflammatory programmed cell death, which can effectively kill malignant cells and enhance anti-tumor immunity (11, 12). Pyroptosis is primarily characterized by DNA fragmentation, chromatin condensation, cell swelling and leakage of cell contents, and has been shown to be related to malignant tumors and inflammatory diseases (13–16). As previous studies showed that compared with apoptosis, pyroptosis may be a more relevant approach to cancer treatment, because it can trigger an anti-tumor immune response and use the patients' own immune systems to specifically recognize and kill tumor cells (17, 18). This study aimed to identify new biomarkers for prognostic prediction of patients with oral squamous cell carcinoma by studying pyroptosis-related molecules.

The expression changes of long non-coding RNAs (lncRNAs) may affect the occurrence and development of tumors (19), and their abnormal expression can also regulate the proliferation, apoptosis and migration of tumor cells, which means that lncRNAs may be served as important biomarkers and therapeutic targets (20).

Although many subjects have studied the relationship between lncRNAs and OSCC (21–23), the relationship between pyroptosis-related lncRNAs and oral squamous cell carcinoma has not yet been fully studied.

In this study, we used the Cancer Genome Atlas (TCGA) database to evaluate the potential value of pyroptosis-related lncRNAs as prognostic tools for OSCC patients. Through TCGA data analysis, 8 pyroptosis-related lncRNAs were determined to be related to OSCC patients' overall survival (OS). And it was proved that their predictive performance for the prognosis of OSCC patients was higher than that of clinicopathological characteristics (age, gender, grade, T stage, N stage). This study proved that pyroptosis-related lncRNAs could be used as biomarkers to predict the prognosis of OSCC patients.

## Materials and methods

### Data acquisition of pyroptosis-related lncRNAs in OSCC patients

Next-generation sequencing (NGS) is a technology that allows thousands to billions of DNA fragments to be sequenced independently at the same time, also known ashigh-throughput or massively parallel sequencing (24). In this study, the RNA-seq data and clinicopathological data of OSCC patients are from TCGA (https://portal.gdc.cancer.gov/). And we use Perl code to organize clinical information. The pyroptosis gene set (Systematic name: M41804) is obtained from the Molecular Signatures Database in Gene Set Enrichment Analysis (http://www.gsea-msigdb.org/gsea/index.jsp).

Spearman correlation analysis of the expression of lncRNAs and pyroptosis genes through the “limma” (https://bioconductor.org/packages/limma/; version 3.14) package of the R software (version 4.1.2), and lncRNAs with high correlation (CorFilter > 0.4; *P* < 0.001) with pyroptosis genes were identified as pyroptosis-related lncRNAs.

### Identification of a prognostic multiple pyroptosis-related lncRNAs signature

We used univariate Cox regression analysis to investigate the pyroptosis-related lncRNAs associated with the prognosis of patients with OSCC (*P* < 0.01). Then, we used Least Absolute Shrinkage method and Selection Operator (LASSO) Cox regression analysis to optimize the prognosis of pyroptosis-related multi-lncRNA signature. In this study, the expression of pyroptosis-related lncRNAs in OSCC patients were substituted into the Cox model, and then the LASSO Cox regression coefficient was used to calculate the risk score of each OSCC patient. Then, we based on the median risk score, the patients were divided into two groups: high-risk group and low-risk group. The Kaplan-Meier method and log-rank test were used to determine the differences in OS between OSCC patients in high-risk group and low-risk group. We used images to visualize the risk score distributions, survival status curves, and expression profiles of prognostic lncRNAs in OSCC patients. Statistical calculations and data plotting were performed through R software (version 4.1.2; R foundation).

### The independence of pyroptosis-related multi-lncRNA signature in predicting the prognosis of OSCC patients

We used R software to determine the independent association of risk scores based on pyroptosis-related multi-lncRNA signature and clinicopathological factors (age, gender, pathological stage and TNM stage) with the prognosis of OSCC patients through univariate and multivariate Cox regression analysis. In addition, we used the “survivalROC” package in R software (version 4.1.2; R foundation) to establish receiver operating characteristic (ROC) curves, and then the ROC curves were used to evaluate the accuracy of these factors (pyroptosis-related multi-lncRNA signature age, gender, pathological stage and TNM stage) in predicting the prognosis of OSCC.

The Kruskal-Wallis and Dunn's *post hoc* test was used to further investigate the association between the expression levels of the eight pyroptosis-related lncRNAs and the clinicopathological characteristics of OSCC patients.

### Principal component analysis (PCA) and gene set enrichment analysis (GSEA)

We used Principal component analysis to detect the differences between patients in low-risk group and high-risk group. And used GSEA (https://www.gsea-msigdb.org/gsea/index.jsp) to study the functions of pyroptosis-related genes in low-risk group and high-risk group. Statistical calculations and data plotting were performed through R software (version 4.1.2; R foundation).

### Reverse verification of clinical OSCC specimens by reverse transcription-quantitative PCR (RT-qPCR)

In order to further validate establishment of the an pyroptosis-related long non-coding RNA signature, we used RT-qPCR to detect the expression levels of 8 pyroptosis-related lncRNAs in clinical OSCC samples. Tumor samples were collected from 41 patients (34 males and 7 females; median age 61 years; age range 42–88 years) with OSCC during surgical procedures between January 2017 and December 2020 (Table 1). These were provided by the Department of Oral and Maxillofacial Surgery, the First Affiliated Hospital of Nanchang University. The study was conducted with prior approval of the first affiliated hospital of Nanchang university ethics committee (Permit No. 2020054), and all patients signed an informed consent form.

**Table 1:** Characteristics of the patients.

	*n* = 41
Age (years):	
≤61	22
>61	19
Sex:	
Male	34
Female	7
Tumour size (cm):	
≤2	0
>2, ≤4	13
>4	28
Differentiation:	
Well	15
Moderate/low	26
Cervical lymph node metastasis:	
Yes	14
No	27
Clinical Staging:	
II	11
III	21
IV	9

Total RNA was extracted from tissues using Trizol reagent (Kangwei Century, CW0580S, CWBIO). Subsequently, a reverse transcription kit (Guangzhou Ribo Biological Co., Ltd.) was used to synthesize cDNA. The mixture was prepared according to the instructions of Takara TB Green Premix Ex Taq IIRT-PCR Kit. The RT-qPCR analysis was performed on Step One Plus Real-Time PCRSystem. The 2-*ΔΔ*CT method was used to calculate the related lncRNA expression level, and the related GAPDH mRNA expression was used as an endogenous control. The primers used are shown in Additional file 1: **Supplementary Table S1**.

### Immune infiltration analysis by single sample gene set enrichment analysis (ssGSEA)

The immune signature score was calculated using the single-sample gene set enrichment analysis (ssGSEA) performed by the GSVA package (Version 1.34.0) in R (version 4.1.2; R foundation). Based on the characteristic genes of 24 different immune cell types, the relative immune cell tumor infiltration level was quantified from the gene expression profiles of each tumor sample. Spearman correlation analysis was used to analyze the correlation between eight pyroptosis-related lncRNAs and the level of immune cell infiltration.

### Statistical analysis

We used SPSS Statistics software (version 25) and R software (version 4.1.2; R foundation) to calculate statistical differences. All experiments were repeated 3 times. The regression analysis of univariate and multivariate Cox proportional hazards analysis uses R software (version 4.1.2; R foundation). For nonparametric analysis, Kruskal-Wallis with Dunn's post-hoc test was used. The Kaplan-Meier method was used to draw the survival curve, and the difference was analyzed by log-rank test. All results are presented as hazard ratios and corresponding 95% confidence interval (CI) and *P* < 0.05 was considered statistically significant.

## Results

### Pyroptosis-related lncRNAs and clinicopathological data in patients with OSCC

**Figure 1:**
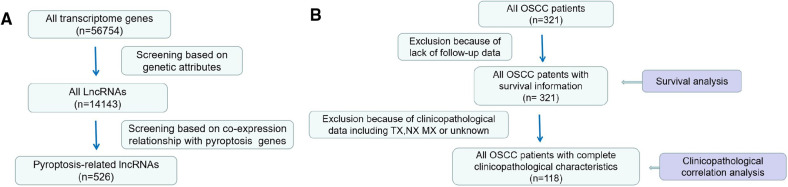
Processes of data selection. (**A**) lncRNA data (**B**) Screening process of clinical pathology data.

We identified a total of 526 lncRNAs associated with pyroptosis, 321 patients with OSCC had survival information, and 118 patients with OSCC had complete clinicopathological information (Figure 1).

### Identification of a prognostic pyroptosis-related lncRNAs signature in OSCC patients

**Figure 2:**
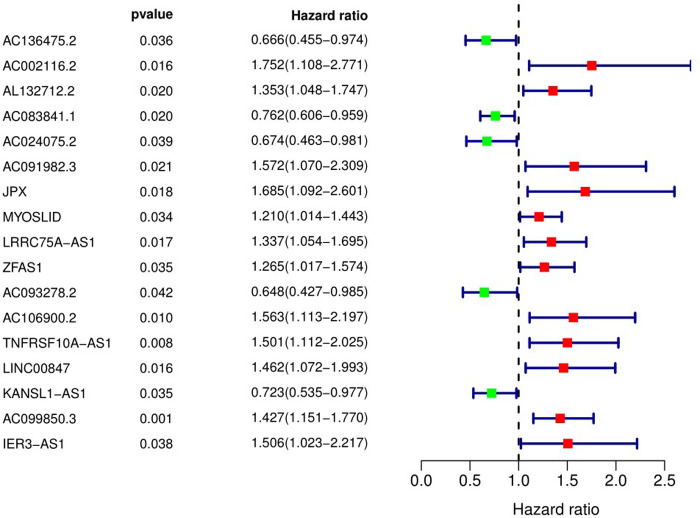
Pyroptosis-related long non-coding RNAs affecting overall survival of patients with OSCC. 95% CI, 95% confidence interval.

We used univariate Cox regression analysis, and identified 17 pyroptosis-related lncRNAs associated with OS in OSCC patients (Figure 2).

We used LASSO Cox regression analysis to further screen the above 17 pyroptosis-related lncRNAs. Among these, eight lncRNAs were incorporated into the Cox model, including AC136475.2, AC024075.2, JPX, ZFAS1, TNFRSF10A-AS1, LINC00847, AC099850.3 and IER3-AS1.

Subsequently, we integrated the expression levels of the above 8 pyroptosis-related lncRNAs and used LASSO Cox regression coefficients to establish a prognostic pyroptosis-related lncRNAs signature in OSCC patients (Table 2). Then the risk scores of OSCC patients were calculated (the 321 patients, excluded 19 patients whose target lncRNA expression is not detected; *n* = 302), and the 302 patients were divided into high-risk group (*n* = 151) and low-risk group (*n* = 151) according to the median value (1.1552).

**Table 2:** The LASSO Cox proportional hazard model of the 8 pyroptosis-related lncRNAs.

id	Coefficient	Hazard ratio	95% Confidence interval	*P*-value
AC136475.2	−0.8679	0.4198	0.2770–0.6364	0
AC024075.2	−0.5774	0.5614	0.3710–0.8494	0.0063
JPX	0.5188	1.68	0.9644–2.9268	0.047
ZFAS1	0.241	1.2725	0.9937–1.6295	0.0461
TNFRSF10A-AS1	0.3176	1.3739	0.9723–1.9413	0.0072
LINC00847	0.5787	1.7838	1.2179–2.6125	0.003
AC099850.3	0.3067	1.3589	1.0828–1.7054	0.0081
IER3-AS1	0.4807	1.6172	1.1018–2.3738	0.0141

**Figure 3:**
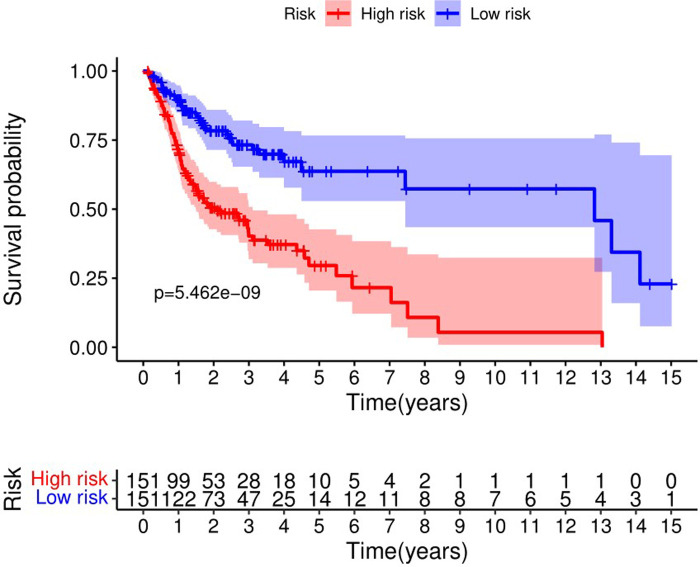
Kaplan-Meier curve showed that the overall survival of the high-risk group was significantly lower than that of the low-risk group.

**Figure 4:**
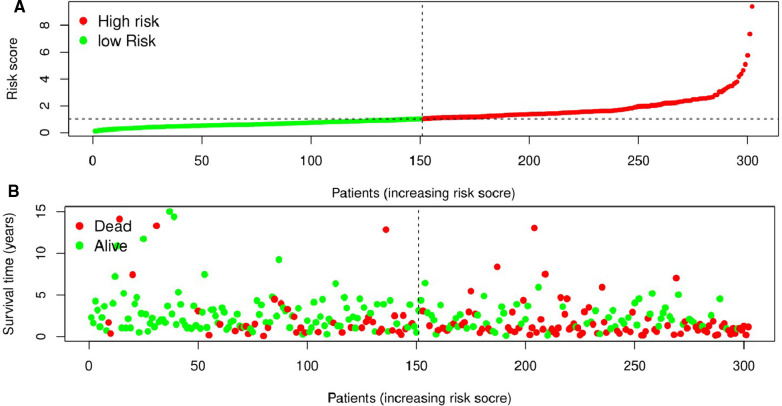
Prognostic value of the eight pyroptosis-related lncRNAs signature in patients with oral squamous cell carcinoma. (**A**) According to the median risk score, patients were divided into low-risk group and high-risk group. And the risk distribution diagram of patients wew arranged by risk score. (**B**) The survival status distribution shows that the mortality rate of patients in the high-risk group is significantly higher than that of patients in the low-risk group, and the survival time is shorter.

**Figure 5:**
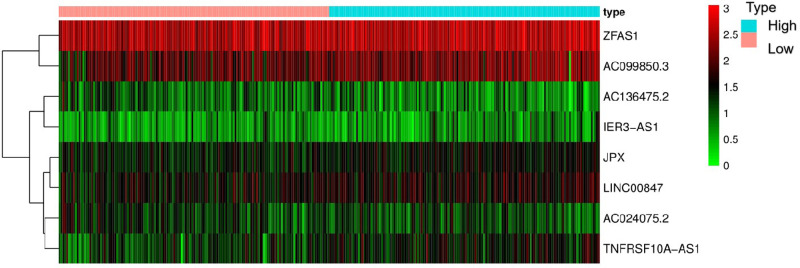
Heat map of the eight pyroptosis-related lncRNA. The expression levels of ZJPX, ZFAS1, TNFRSF10A-AS1, LINC00847, AC099850.3 and IER3-AS1 in high-risk patients were higher than those in low-risk patients. The expression levels of AC136475.2 and AC024075.2 in high-risk patients were lower than those in low-risk patients.

The Kaplan-Meier curve showed that the OS of the high-risk group was significantly lower than that of the low-risk group (*P* = 5.462 × 10^9^; Figure 3). The distribution of risk scores, survival status, and expression profiles of 8 prognostic pyroptosis-related lncRNAs of OSCC patients are shown in Figures 4, 5. The results showed that the mortality rate of OSCC patients with high-risk scores was higher than that of OSCC patients with low-risk scores. The expression levels of ZJPX, ZFAS1, TNFRSF10A-AS1, LINC00847, AC099850.3 and IER3-AS1 in high-risk patients with OSCC were higher than those in low-risk patients with OSCC. The expression levels of AC136475.2 and AC024075.2 in high-risk patients with OSCC were lower than those in low-risk patients with OSCC.

### The relationship between the feature of eight pyroptosis-related lncRNAs and the prognosis of patients with OSCC

**Figure 6:**
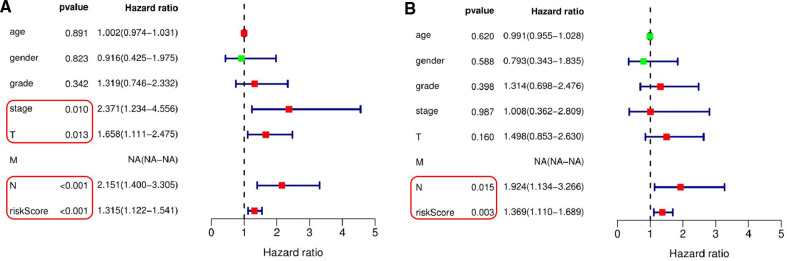
Independent testing of the association between the eight pyroptosis-related lncRNAs signature and prognosis of patients. (**A**) Univariate Cox regression analysis showed that the signature based on risk score (*P* < 0.001), T stage (*P* = 0.013), N stage (*P* < 0.001) and TNM comprehensive stage (*P* = 0.010) were significantly associated with patient survival. (**B**) Multivariate Cox regression analysis demonstrated that the signature based on risk score (*P* = 0.003) and N stage (*P* = 0.015) were independent factors associated with patient survival.

The results of univariate Cox regression analysis showed that the risk score based on 8 pyroptosis-related lncRNAs feature, the patients' T stage, N stage, and TNM comprehensive stage were significantly correlated with the patients’ survival rate (Figure 6A). In addition, the multivariate Cox regression analysis showed that the risk score based on 8 pyroptosis-related lncRNAs feature and the patients’ N stage were independent factors related to OSCC patients with OS (Figure 6B). Due to the lack of M1 in the case data, the effective value of M staging cannot be obtain.

**Figure 7:**
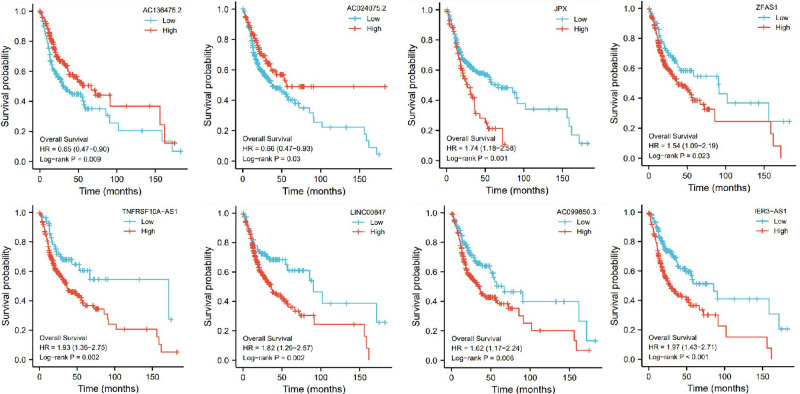
The influence of eight genes (AC136475.2, AC024075.2, JPX, ZFAS1, TNFRSF10A-AS1, LINC00847, AC099850.3, IER3-AS1) on survival probability.

In addition, based on the TCGA genome database, we used R software to analyze the association of these 8 pyroptosis-related genes with the prognosis of OSCC patients. These genes were found to be associated with the prognosis of OSCC patients, and the groups with high expression of ZJPX, ZFAS1, TNFRSF10A-AS1, LINC00847, AC099850.3 and IER3-AS1 had a worse prognosis, the groups with low expression of AC136475.2 and AC024075.2 had a worse prognosis (Figure 7).

### The receiver operating characteristic (ROC) curves for the 8 pyroptosis-related lncRNAs

**Figure 8:**
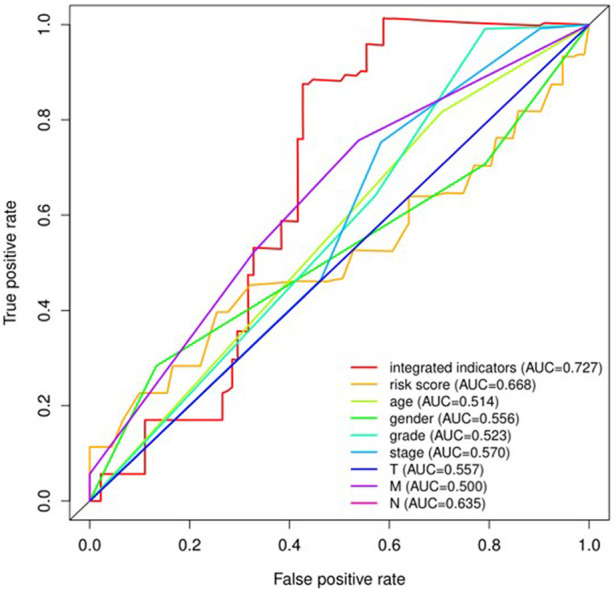
The receiver operating characteristic (ROC) curve analysis of risk scoreof the eight pyroptosis-related lncRNAs and other clinicopathological characteristics including age, gender, grade, T stage, M stage, N stage, and TNM comprehensive stage for predicting the overall survival of patients with oral squamous cell carcinoma. AUC, area under the curve.

**Figure 9:**
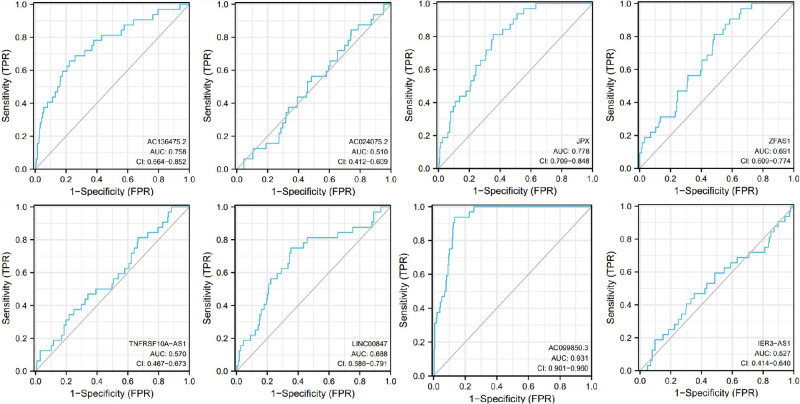
Receiver operating characteristic curves (ROC) for the AC136475.2, AC024075.2, JPX, ZFAS1, TNFRSF10A-AS1, LINC00847, AC099850.3, IER3-AS in the patients with oral squamous cell carcinoma. AUC, area under the curve.

The receiver operating characteristic (ROC) curves for 8 pyroptosis-related lncRNAs are shown in Figures 8, 9. The ROC area for risk score was 0.668, which was higher than that of the other clinicopathological characteristics. The ROC area for age, gender, grade, T stage, M stage, N stage, and TNM comprehensive stage were 0.514, 0.556, 0.523, 0.557, 0.500, 0.635 and 0.570 (Figure 8). And we integrated clinical factors and risk score into integrates indicators, the ROC area for integrates indicators was 0.727. Moreover, the area values under the ROC curve of each gene of these 8 pyroptosis-related lncRNAs was between 0.5 and 1 (Figure 9), indicating that the accuracy of using these genes for disease diagnosis and predicting the overall survival rate of patients is very high.

### Relationship between eight pyroptosis-related lncRNAs and clinicopathological characteristics in patients with OSCC

**Figure 10:**
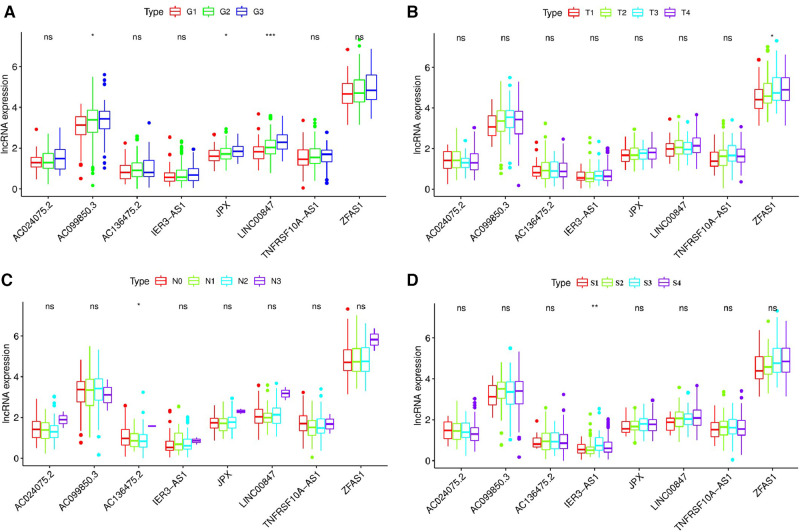
Relationship between the expression levels of eight pyroptosis-related lncRNAs and the clinicopathological characteristics of patients with OSCC. (**A**) JPX (*P* < 0.05), AC099850.3 (*P* < 0.05) and LINC00847 (*P* < 0.001) were significantly correlated with the pathological grade of OSCC patients. (**B**) ZFAS1 (*P* < 0.05) was significantly correlated with T stage of OSCC patients (**C**) AC136475.2 (*P* < 0.05) was significantly correlated with N stage of OSCC patients. (**D**) IER3-AS1 (*P* < 0.01) was significantly correlated with clinical stage of OSCC patients. G1, well differentiated; G2, moderately differentiated; G3, poorly differentiated; G4, undifferentiated; T, T stage; N, N stage; S, TNM synthesis stage; S1, first stage; S2, second stage; S3, third stage; S4, fourth stage. *P < 0.05; **P < 0.01; ***P < 0.001; ns, not significant.

We used the Kruskal-Wallis test to investigate the relationship between the expression levels of 8 pyroptosis-related lncRNAs and the clinicopathological characteristics of patients with OSCC (Figure 10). The results showed that JPX (*P* < 0.05), AC099850.3 (*P* < 0.05), LINC00847 (*P* < 0.001) were significantly correlated with the pathological grade of OSCC patients (Figure 10A), ZFAS1 (*P* < 0.05) was significantly correlated with T stage of OSCC patients (Figure 10B), AC136475.2 (*P* < 0.05) was significantly correlated with N stage of OSCC patients (Figure 10C), and IER3-AS1 (*P* < 0.01) was significantly correlated with clinical stage of OSCC patients (Figure 10D).

### Pyroptotic status of low-risk groups and high-risk groups

**Figure 11:**
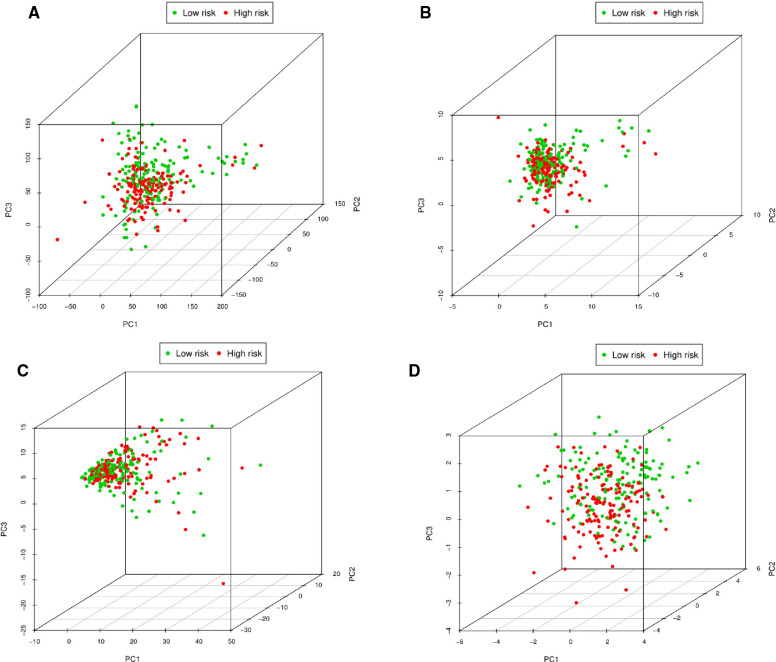
Principal component analysis between low-risk and high-risk patients. (**A**) PCA of expression differences of all genes between low-risk and high-risk OSCC patients. (**B**) PCA of expression differences of pyroptosis-related genes between low-risk and high-risk OSCC patients. (**C**) PCA of expression differences of pyroptosis-related lncRNAs between low-risk and high-risk OSCC patients. (**D**) PCA of expression differences of eight pyroptosis-related lncRNAs between low-risk and high-risk OSCC patients.

PCA was performed to explore the expression differences of all genes, pyroptosis-related genes, pyroptosis-related lncRNAs, and 8 pyroptosis-related lncRNAs between low-risk and high-risk OSCC patients. When we used the expression of all genes as a spatial indicator, the two groups of patients were relatively concentrated. When the indicators were gradually optimized from all genes to pyroptosis-related genes, pyroptosis-related lncRNAs, and 8 pyroptosis-related lncRNAs, the two groups of patients were gradually dispersed (Figure 11). This indicated that the signature established in this study showed better discriminative power in predicting the prognosis of OSCC patients compared with other genetic indicators.

**Figure 12:**
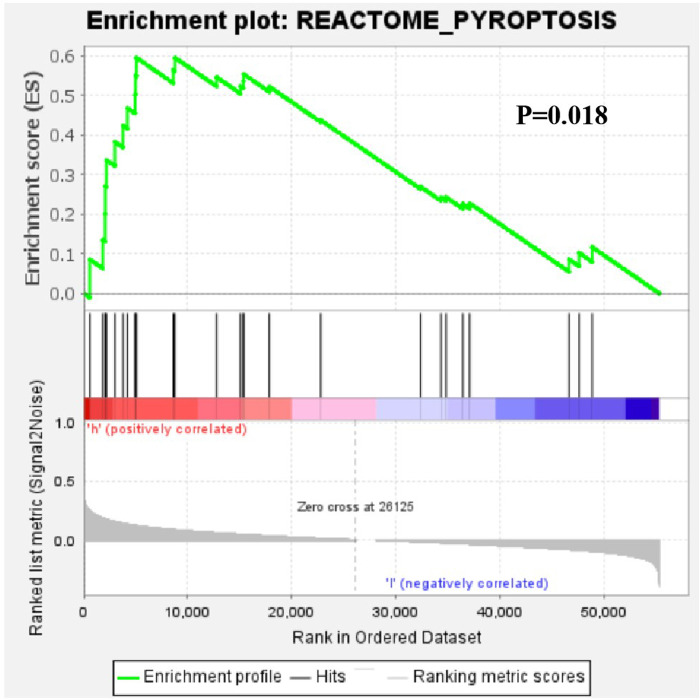
Gene set enrichment analysis of the differential expression of the pyroptosis-related lncRNAs between low-risk and high-risk patients with OSCC.

Further functional annotation with GSEA showed that the difference in gene function were significantly enriched in the gene sets of pyroptosis between low-risk and high-risk patients (Figure 12). It shows that the prognosis of patients can be judged according to the overall increase or decrease of pyroptosis.

### Relationship between the eight pyroptosis-related lncRNAs and immune infiltration level

**Figure 13:**
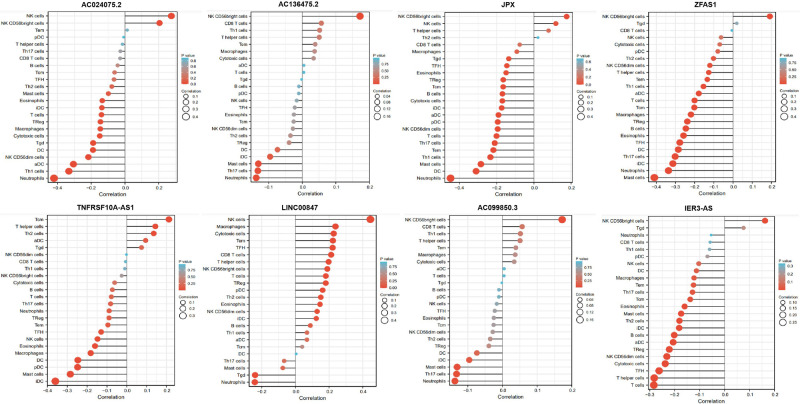
Relationship between 8 pyroptosis-related lncRNAs and immune infiltration. Lollipop plot shows the correlation between AC136475.2, AC024075.2, JPX, ZFAS1, TNFRSF10A-AS1, LINC00847, AC099850.3 and IER3-AS expression and 24 immune cell subsets infiltration. The size of dots indicates the absolute Spearman *r* value.

**Figure 14:**
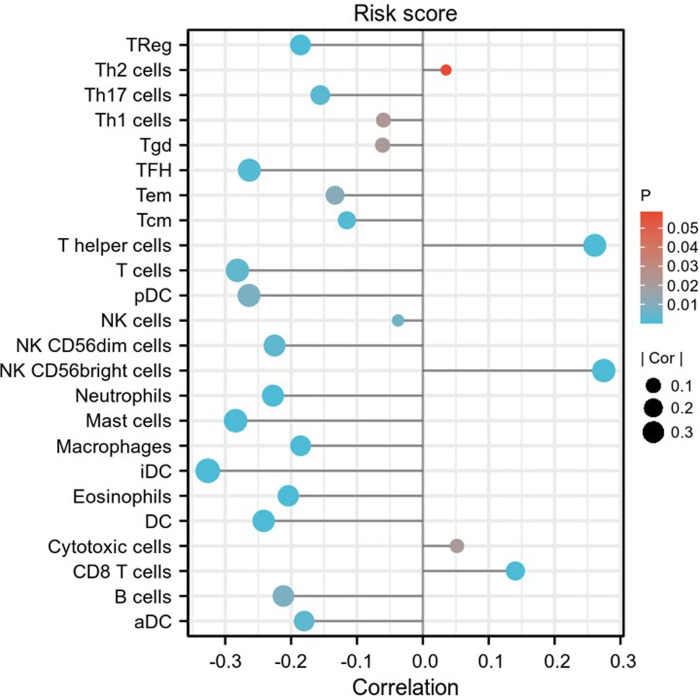
Relationship between risk score and immune infiltration. Lollipop plot shows the correlation between risk score and 24 immune cell subsets infiltration. The size of dots indicates the absolute Spearman r value.

The associations between the expression of the eight pyroptosis-related lncRNAs and the abundance of immune cells infiltration were analyzed using ssGSEA. As illustrated in the lollipop plot **(**Figures 13, 14), this modle, and expression of AC136475.2, AC024075.2, JPX, ZFAS1, TNFRSF10A-AS1, LINC00847, AC099850.3, IER3-AS in this model exhibited a strong positive correlation with some immunocytes, including macrophages, T cells, B cells, Th1 cells, Th2 cells, and NK cells, etc. These findings suggest that these pyroptosis-related lncRNAs affect immune responses by influencing the infiltration of immunocytes into the microenvironment of OSCC.

### RT-qPCR to verify the effect of pyroptosis-related genes on patient survival

**Figure 15:**
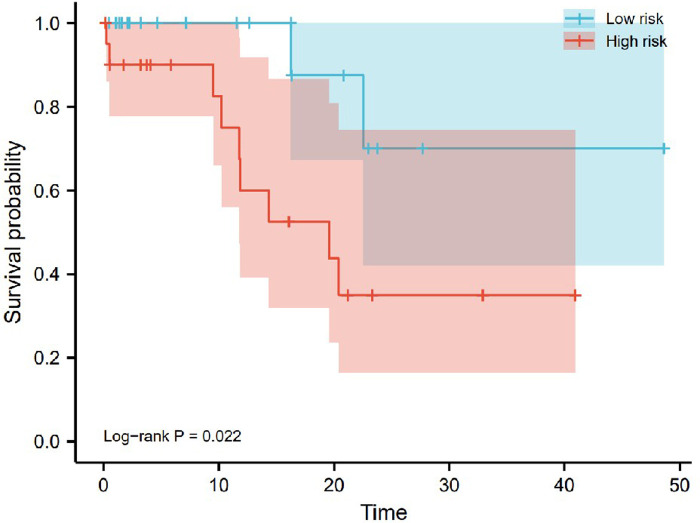
Kaplan-Meier analysis of overall survival plots based on RT-qPCR analysis of 41 OSCC patients. The overall survival of the high-risk group was significantly lower than that of the low-risk group (*P* = 0.022).

To evaluate the reliability of the bioinformatics results, we used RT-qPCR to detect the expression levels of 8 pyroptosis-related lncRNAs in tumor tissues of 41 OSCC patients. Patients were divided into low-risk and high-risk groups based on RT-qPCR and signature risk scores. And we used Kaplan-Meier analysis to analyze OS of the low-risk group and the high-risk group, showed that the OS of the low-risk group was significantly higher than that of the high-risk group (*P* = 0.022; Figure 15).

## Discussion

Cell necrosis, apoptosis, necroptosis, autophagy, ferroptosis, and pyroptosis all belong to programmed cell death (PCD) (25). Pyroptosis is also an inflammatory form of PCD (26). There is increasing evidence that pyroptosis affects tumor proliferation, invasion, and metastasis (27). Furthermore, pyroptosis may be able to the function as a form of immunogenic cell death (ICD) and may act synergistically with cancer immunotherapy (26). Therefore, pyroptosis may be a double-edged sword for the development of tumors, which requires further in-depth research.

Pyroptosis promotes inflammatory cell death of cancer cells, and thus decreased expression of some pyroptotic inflammasomes was found in cancer cells (15, 28). Recent studies have reported that some molecules affect the pyroptotic inflammasome and promote pyroptosis (27–28). These molecules, including some noncoding RNAs, hold promise as targets for effective treatment of different cancers (29). It has been reported that lncRNA NEAT1 modulated ionizing radiation that induced pyroptosis and viability in human colorectal cancer cells by regulating the expression of miR-448 (30). This study provides important insights into the potential role of lncRNA NEAT1 in ionizing radiation-induced pyroptosis mechanistic insights. Therefore, studying and utilizing the roles of lncRNAs in the regulation of pyroptosis will improve the clinical diagnostic accuracy and therapeutic effect of tumors.

This study used the TCGA dataset to investigate the predictive value of pyroptosis-related lncRNAs on the prognosis of oral squamous cell carcinoma. We identified 17 pyroptosis-related lncRNAs associated with OS in OSCC patients by univariate Cox regression analysis. Subsequently, 8 pyroptosis-related lncRNAs that significantly affected the prognosis of OSCC patients were identified by LASSO Cox regression analysis and Kaplan-Meier method, including AC136475.2, AC024075.2, JPX, ZFAS1, TNFRSF10A-AS1, LINC00847, AC099850. 3 and IER3-AS1. Risk signatures were determined based on these pyroptosis-related lncRNAs, which could classify OSCC cancer patients into high- and low-risk groups. Our results showed that the high-risk group had shorter OS than the low-risk group. As described in the results, among the 8 pyroptosis-related lncRNAs, 2 lncRNAs are protective factors (AC136475.2 and AC024075.2), and the remaining 6 lncRNAs are risk factors (JPX, ZFAS1, TNFRSF10A-AS1, LINC00847, AC099850.3 and IER3-AS1). And the association between the expression of these pyroptosis-related lncRNAs and the abundance of immune cell infiltration was analyzed by ssGSEA. It was found that these pyroptosis-related lncRNAs could affect the infiltration of immune cells into the OSCC microenvironment. This study identified pyroptosis-related lncRNA signature with satisfactory performance.

In addition to our exploration of these pyroptosis-related lncRNAs, other studies have also investigated the related roles of the above lncRNAs.

AC136475.2 has not previously reported its effect in patients with head and neck squamous cell carcinoma. Ma et al (31) established a risk score model based on this gene for breast cancer patients, demonstrating that the gene can be used as a predictor of breast cancer survival and may be a potential therapeutic target for breast cancer.

Related studies of AC024075.2 have not been reported.

A study (32) has reported that LncRNA JPX is highly expressed in OSCC cells. Silencing lncRNA JPX could inhibit the proliferation, migration and invasion of OSCC cells. And the lncRNA JPX could bind to miR-944 and then enhances N-cadherin (CDH2) through a competing endogenous RNA (ceRNA) mechanism. In short, lncRNA JPX promotes the proliferation, migration and invasion of OSCC cells through the miR-944/CDH2 axis, which provides a new potential targeted therapy direction for OSCC.

Accumulating evidence indicates that long non-coding RNA zinc finger antisense 1 (ZFAS1) is a novel lncRNA with potential functions in human cancer pathology and physiology (33, 34), which is involved in tumorigenesis and tumor progression (35). Feng et al. showed that the expression of ZFAS1 is related to the chemosensitivity and prognosis of cervical cancer and is involved in the progression of cervical cancer (36).

Ren et al reported that RP11-1149O23.2 has a high coexpression coefficient of 0.81 with TNFRSF10A antisense RNA 1 (TNFRSF10A-AS1), suggesting the possibility of RP11-1149O23.2 in cis-regulation of TNFRSF10A. And RP11-1149O23.2 is localized at the minus chain relative to the protein-coding gene TNFRSF10A, the protein of TNFRSF10A is a apoptosis-promoting receptor to mediate the extrinsic apoptotic pathways. In addition, RP11-1149O23.2 is significantly associated with the overall survival of patients with gastric cancer (37).

LINC00847 located at 5q35.3, is a newly discovered lncRNA. In recent years, aberrant expression of LINC00847 has been reported in various tumors including renal cell carcinoma, breast cancer and lung cancer (38–40). Huan et al showed that LINC00847 could increase IFITM1 expression by sponging miR-147a, thereby exhibiting tumor-promoting effects on non-small cell lung cancer progression (41).

AC099850.3 is a recently discovered lncRNA. Zhong et al were the first to identify AC099850.3 as a prognosis-related lncRNA by TCGA database, and they further through *in vitro* and *in vivo* experiments to clarify the role of AC099850.3 in patients with hepatocellular carcinoma. The results showed that AC099850.3 was highly expressed in hepatocellular carcinoma cell lines and tissues, and was related to poor prognosis in patients with hepatocellular carcinoma. And AC099850.3 significantly promoted migration, invasion and proliferation of hepatocellular carcinoma cell through the PRR11/PI3K/AKT pathway (42). Jiang et al revealed that AC099850.3 was associated with autophagy and it could predict OS in patients with OSCC (43).

Immediate early response gene 3 (IER3), also known as IEX-1, gly96, Dif-2, PRG-1 or p22, is a member of the NUPR1/RELB/IER3 survival pathway. Hamid et al revealed that NUPR1/RELB/IER3 pathway was required for oncogenic-dependent transformation of the pancreas (44). Jin et al showed that etoposide induced death of cervical cancer cells through the c-Abl/TAp73/IER3 signaling axis (45).

To our knowledge, in addition to AC024075.2, seven other lncRNAs have been reported that they were correlated with the growth and prognosis of some tumors, therefore we predicted that these lncRNAs could also represent potential targets for the treatment of OSCC.

In recent years, with the progress of research, it has been gradually recognized that tumor progression is closely related to changes in its immune microenvironment. At present, the development and application of immunotherapy for oral cancer is relatively slow. Therefore, we explored the link between target lncRNAs and immune cell infiltration. And we found that the target lncRNA is closely related to immune cell infiltration, which may provide a theoretical basis for the study of oral cancer immunotherapy.

Our current study still has some limitations. This study utilized data from high-throughput RNA-sequencing for analysis, and although we used clinical 41 samples for validation, lacked validation in large clinical samples. Moreover, the roles of the eight pyroptosis-related lncRNAs are worthy of continued *in vitro* and *in vivo* studies.

## Conclusion

In this study, 8 pyroptosis-related lncRNAs were identified to be associated with OS in OSCC patients through TCGA data analysis. Risk signatures were determined based on these pyroptosis-related lncRNAs, which could classify OSCC patients into high- and low-risk groups. And risk signatures could predict patients at higher risk of death, therefore better treatment should be given to higher-risk patients.

## Data Availability

The original contributions presented in the study are included in the article/**Supplementary Material**, further inquiries can be directed to the corresponding author/s.

## References

[1] Siegel RL Miller KD Jemal A. Cancer statistics. CA Cancer J Clin. (2018) 68(1):7–30. 10.3322/caac.21442 29313949

[2] Jiawei Y Hongyan X. Regulation of transforming growth factor-beta1 by circANKS1B/miR-515-5p affects the metastatic potential and cisplatin resistance in oral squamous cell carcinoma. Bioengineered. (2021) 12(2):12420–30. 10.1080/21655979.2021.2005221 34781814 PMC8810104

[3] Smittenaar CR Petersen KA Stewart K Moitt N. Cancer incidence and mortality projections in the UK until 2035. Br J Cancer. (2016) 115(9):1147–55. 10.1038/bjc.2016.304 27727232 PMC5117795

[4] Zihang L Bin C Xiaoan T. Epithelial-to-mesenchymal transition in oral squamous cell carcinoma: challenges and opportunities. Int J Cancer. (2021) 148(7):1548–61. 10.1002/ijc.33352 33091960

[5] Montero PH Patel SG. Cancer of the oral cavity. Surg Oncol Clin N Am. (2015) 24(3):491–508. 10.1016/j.soc.2015.03.006 25979396 PMC5018209

[6] Santos HB dos Santos TK Paz AR Cavalcanti YW Nonaka CF Pina G Clinical findings and risk factors to oral squamous cell carcinoma in young patients: a 12-year retrospective analysis. Med Oral Patol Oral Cir Bucal. (2016) 21(2):e151–6. 10.4317/medoral.20770 26827057 PMC4788792

[7] Yete S D'Souza W Saranath D. High-risk human papillomavirus in oral cancer: clinical implications. Oncology. (2018) 94(3):133–41. 10.1159/000485322 29241220

[8] Yangyang S Xiaolin N Min Z Menglin W. Epstein-Barr virus infection and oral squamous cell carcinoma risk: a meta-analysis. PLoS One. (2017) 12(10):e0186860. 10.1371/journal.pone.0186860 29065191 PMC5655447

[9] Mohd Bakri M Mohd Hussaini H Rachel Holmes A David Cannon R Mary Rich A. Revisiting the association between candidal infection and carcinoma, particularly oral squamous cell carcinoma. J Oral Microbiol. (2010) 2:5780. 10.3402/jom.v2i0.5780 PMC3084579 21523221

[10] Falzone L Lupo G La Rosa GRM Crimi S Anfuso CD. Identification of novel MicroRNAs and their diagnostic and prognostic significance in oral cancer. Cancers (Basel). (2019) 11(5):610. 10.3390/cancers11050610 PMC6562527 31052345

[11] Kewei W Xuan X Maolin J Jisi L Jielian Z Yuan Y. An NIR-fluorophore-based theranostic for selective initiation of tumor pyroptosis-induced immunotherapy. Small. (2021) 17(36):e2102610. 10.1002/smll.202102610 34323375

[12] Bergsbaken T Fink SL Cookson BT. Pyroptosis: host cell death and inflammation. Nat Rev Microbiol. (2009) 7(2):99–109. 10.1038/nrmicro2070 19148178 PMC2910423

[13] Jorgensen I Rayamajhi M Miao EA. Programmed cell death as a defence against infection. Nat Rev Immunol. (2017) 17(3):151–64. 10.1038/nri.2016.147 28138137 PMC5328506

[14] Kovacs SB Miao EA. Gasdermins: effectors of pyroptosis. Trends Cell Biol. (2017) 27(9):673–84. 10.1016/j.tcb.2017.05.005 28619472 PMC5565696

[15] Jianjin S Wenqing G Feng S. Pyroptosis: gasdermin-mediated programmed necrotic cell death. Trends Biochem Sci. (2017) 42(4):245–54. 10.1016/j.tibs.2016.10.004 27932073

[16] Dan W Sheng W Guocan Y Xiaoyuan C. Cell death mediated by the pyroptosis pathway with the aid of nanotechnology: prospects for cancer therapy. Angew Chem Int Ed Engl. (2021) 60(15):8018–34. 10.1002/anie.202010281 32894628

[17] Gajewski TF Woo SR Zha Y Spaapen R Zheng Y Leticia C Cancer immunotherapy strategies based on overcoming barriers within the tumor microenvironment. Curr Opin Immunol. (2013) 25(2):268–76. 10.1016/j.coi.2013.02.009 23579075

[18] Schumacher TN Schreiber RD. Neoantigens in cancer immunotherapy. Science. (2015) 348(6230):69–74. 10.1126/science.aaa4971 25838375

[19] Agirre X Meydan C Jiang Y Garate L Doane AS Akanksha V Long non-coding RNAs discriminate the stages and gene regulatory states of human humoral immune response. Nat Commun. (2019) 10(1):821. 10.1038/s41467-019-08679-z 30778059 PMC6379396

[20] Tianyu L Chen Y Hongbin N Weibang L Huiying Y. Expression of the long non-coding RNA H19 and MALAT-1 in growth hormone-secreting pituitary adenomas and its relationship to tumor behavior. Int J Dev Neurosci. (2018) 67:46–50. 10.1016/j.ijdevneu.2018.03.009 29604339

[21] Lei Z Xiang M Xin Wei Z Deng Cheng Y Ran C Yong J Long non-coding RNAs in Oral squamous cell carcinoma: biologic function, mechanisms and clinical implications. Mol Cancer. (2019) 18(1):102. 10.1186/s12943-019-1021-3 31133028 PMC6535863

[22] Yong D Haiyan Y Yue L Wenli G Tufeng Z Haitao S Long non-coding RNA LINC01137 contributes to oral squamous cell carcinoma development and is negatively regulated by miR-22-3p. Cell Oncol (Dordr). (2021) 44(3):595–609. 10.1007/s13402-021-00586-0 33797737 PMC12980792

[23] Xiaojie L Changhong M Li Z Na L Xiaohong Z Jianya H LncRNAAC132217.4, a KLF8-regulated long non-coding RNA, facilitates oral squamous cell carcinoma metastasis by upregulating IGF2 expression. Cancer Lett. (2017) 407:45–56. 10.1016/j.canlet.2017.08.007 28823965

[24] Gu W Miller S Chiu CY. Clinical metagenomic next-generation sequencing for pathogen detection. Annu Rev Pathol. (2019) 14:319–38. 10.1146/annurev-pathmechdis-012418-012751 30355154 PMC6345613

[25] Bedoui S Herold MJ Strasser A. Emerging connectivity of programmed cell death pathways and its physiological implications. Nat Rev Mol Cell Biol. (2020) 21(11):678–95. 10.1038/s41580-020-0270-8 32873928

[26] Jianglin Z Zijie Z Yue Q Minjie W Hao Y Wu Z A pyroptosis-related gene prognostic index correlated with survival and immune microenvironment in glioma. J Inflamm Res. (2022) 15:17–32. 10.2147/JIR.S341774 35018108 PMC8742621

[27] Yuan F Shengwang T Yutian P Wei L Qiming W. Pyroptosis: a new frontier in cancer. Biomed Pharmacother. (2020) 121:109595. 10.1016/j.biopha.2019.109595 31710896

[28] Guo H Callaway JB Ting JP. Inflammasomes: mechanism of action, role in disease, and therapeutics. Nat Med. (2015) 21(7):677–87. 10.1038/nm.3893 26121197 PMC4519035

[29] Tripathi V Shen Z Chakraborty A Giri S Freier SM Xiaolin W Long noncoding RNA MALAT1 controls cell cycle progression by regulating the expression of oncogenic transcription factor B-MYB. PLoS Genet. (2013) 9(3):e1003368. 10.1371/journal.pgen.1003368 23555285 PMC3605280

[30] Fei S Junzhao D Jie Z Hanjiang F Xiaofei Z. Long non-coding RNA nuclear paraspeckle assembly transcript 1 regulates ionizing radiation-induced pyroptosis via microRNA-448/gasdermin E in colorectal cancer cells. Int J Oncol. (2021) 59(4):79. 10.3892/ijo.2021.5259 34476497 PMC8448542

[31] Wei M Fangkun Z Xinmiao Y Shu G Huandan S. Immune-related lncRNAs as predictors of survival in breast cancer: a prognostic signature. J Transl Med. (2020) 18(1):442. 10.1186/s12967-020-02522-6 33225954 PMC7681988

[32] Yuan Y Shaoshan C Na L Yue Y Zhiwen L. LncRNA JPX overexpressed in oral squamous cell carcinoma drives malignancy via miR-944/CDH2 axis. Oral Dis. (2021) 27(4):924–33. 10.1111/odi.13626 32881231

[33] Changyi F Jianbao Z Yue B Chenchen L Chenglong H Dongwang Y Long non-coding ribonucleic acid zinc finger antisense 1 promotes the progression of colonic cancer by modulating ZEB1 expression. J Gastroenterol Hepatol. (2017) 32(6):1204–11. 10.1111/jgh.13646 27862275

[34] Xiaodi J Zhi Y Zhiwei L. Zinc finger antisense 1: a long noncoding RNA with complex roles in human cancers. Gene. (2019) 688:26–33. 10.1016/j.gene.2018.11.075 30503395

[35] Tao L Junjie X Chuan S Dongfeng C Yuan S Zhichong W Amplification of long noncoding RNA ZFAS1 promotes metastasis in hepatocellular carcinoma. Cancer Res. (2015) 75(15):3181–91. 10.1158/0008-5472.CAN-14-3721 26069248

[36] Lan Lan F Fang Rong S Jin Hua Z You Guo C. Expression of the lncRNA ZFAS1 in cervical cancer and its correlation with prognosis and chemosensitivity. Gene. (2019) 696:105–12. 10.1016/j.gene.2019.01.025 30738960

[37] Wu R Jian Z Wei L Zongcheng L Shuofeng H Jian S A tumor-specific prognostic long non-coding RNA signature in gastric cancer. Med Sci Monit. (2016) 22:3647–57. 10.12659/msm.901190 27727196 PMC5072383

[38] Safarpour-Dehkordi M Doosti A Jami MS. Integrative analysis of lncRNAs in kidney cancer to discover a new lncRNA (LINC00847) as a therapeutic target for staphylococcal enterotoxin tst gene. Cell J. (2020) 22(Suppl 1):101–9. 10.22074/cellj.2020.6996 32779439 PMC7481890

[39] Yuanyuan H Xidong G Yin D Yong S Xiaohong X. Bioinformatics analysis of prognosis-related long non-coding RNAs in invasive breast carcinoma. Oncol Lett. (2020) 20(1):113–22. 10.3892/ol.2020.11558 PMC7285808 32565939

[40] Hui Y Qinghua X Fang L Xun Y Jialei W Meng X. Identification and validation of long noncoding RNA biomarkers in human non-small-cell lung carcinomas. J Thorac Oncol. (2015) 10(4):645–54. 10.1097/JTO.0000000000000470 25590602

[41] Huan L Yao Kai C Qiu W An Qi S Min W Ping H Long non-coding RNA LINC00847 induced by E2F1 accelerates non-small cell lung cancer progression through targeting miR-147a/IFITM1 axis. Front Med (Lausanne). (2021) 8:663558. 10.3389/fmed.2021.663558 33968966 PMC8100058

[42] Fangjing Z Sheng L Donghai H Liwen C. LncRNA AC099850.3 promotes hepatocellular carcinoma proliferation and invasion through PRR11/PI3K/AKT axis and is associated with patients prognosis. J Cancer. (2022) 13(3):1048–60. 10.7150/jca.66092 35154469 PMC8824888

[43] Qingkun J Danfeng X Fanzhe S Jiaxuan Q. Prognostic significance of an autophagy-related long non-coding RNA signature in patients with oral and oropharyngeal squamous cell carcinoma. Oncol Lett. (2021) 21(1):29. 10.3892/ol.2020.12290 33240435 PMC7681235

[44] Hamidi T Algül H Cano CE Sandi MJ Molejon MI Riemann M Nuclear protein 1 promotes pancreatic cancer development and protects cells from stress by inhibiting apoptosis. J Clin Invest. (2012) 122(6):2092–103. 10.1172/JCI60144 22565310 PMC3366404

[45] Jin H Suh DS Kim TH Yeom JH Lee K Bae J. IER3 Is a crucial mediator of TAp73*β*-induced apoptosis in cervical cancer and confers etoposide sensitivity. Sci Rep. (2015) 5:8367. 10.1038/srep08367 25666857 PMC4322356

